# Hypoperfusion cerebral infarction after carotid artery stenting: A case report

**DOI:** 10.3389/fsurg.2022.1077826

**Published:** 2023-01-09

**Authors:** Yuerong Ma, Renwei Zhang, Yumin Liu

**Affiliations:** Department of Neurology, Zhongnan Hospital of Wuhan University, Wuhan, China

**Keywords:** acute cerebral infarction, cerebral artery stenosis, carotid artery stent implantation, hypoperfusion syndrome, hyperperfusion syndrome

## Abstract

Carotid artery stent implantation (CAS) plays an important role in preventing cerebral infarction associated with carotid stenosis. The postoperative complications of CAS include cerebral hyperperfusion syndrome (CHS), cerebral infarction, vascular injury, carotid sinus reaction, and stent restenosis. Hyperperfusion syndrome (CHS) is a serious complication that arises after the performance of carotid endarterectomy (CEA) or CAS and is characterized by high blood pressure, headache, epilepsy, and focal neurological deficit. Therefore, it is very important to evaluate and diagnose CHS. Cerebral infarction after CAS is often caused by distal embolism due to the shedding of microemboli. With the application of distal brain protection devices, the risk of distal embolism is significantly reduced. In this study, we report a rare case of hypoperfusion cerebral infarction after carotid artery stenting in a patient with severe carotid stenosis complicated with contralateral common carotid artery occlusion.

## Introduction

Carotid artery stent implantation (CAS) plays an essential role in preventing cerebral infarction associated with carotid stenosis. The postoperative complications of CAS include cerebral hyperperfusion syndrome (CHS), cerebral infarction, vascular injury, carotid sinus reaction, and stent restenosis. Hyperperfusion syndrome (CHS) is a severe complication that arises after the performance of carotid endarterectomy (CEA) or CAS and is characterized by high blood pressure, headache, epilepsy, and focal neurological deficit. Therefore, it is essential to evaluate and diagnose CHS. Cerebral infarction after CAS is often caused by distal embolism due to the shedding of microemboli. With the application of distal brain protection devices, the risk of distal embolism is significantly reduced. In this study, we report a rare case of hypoperfusion cerebral infarction after carotid artery stenting in a patient with severe carotid stenosis complicated with contralateral common carotid artery occlusion.

## Case presentation

**Figure 1:**
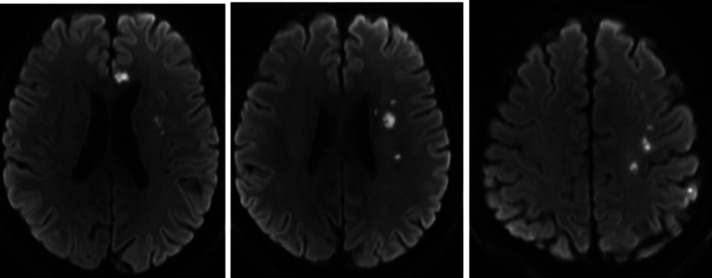
Admission brain magnetic resonance imaging showing acute cerebral infarction.

**Figure 2:**
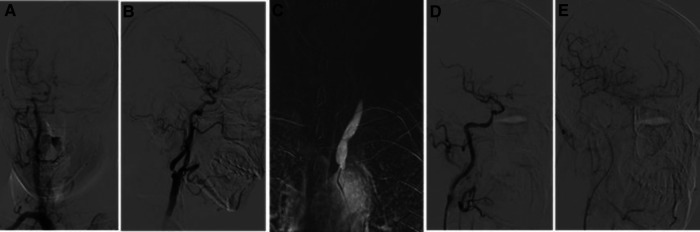
Digital subtraction angiography (DSA) image after admission. (**A**) DSA from the right common carotid artery showing that the anterior communication is open and compensated from right to left. (**B**) Severe stenosis of the C1 segment of the right internal carotid artery. (**C**) DSA of the left common carotid artery showing an occlusion of the common carotid artery. (**D,E**) Postoperative angiography of the right CAS showing an improvement of right to left blood flow compensation. CAS, carotid artery stent implantation.

A 59-year-old male patient was admitted to the hospital on May 22, 2021, because of a sudden right limb weakness for more than 2 days. Before this 2-day period, the patient had no signs of obvious inducement to develop any symptoms of weakness in the right upper and lower limbs. The patient had a long-term history of hypertension with poor blood pressure control and that of smoking and drinking. A neurological examination showed that the muscle strength of the right upper and lower limbs was slightly poor. The patient’s National Institutes of Health Stroke Scale (NIHSS) score was 0. A computed tomography angiography (CTA) of the external hospital showed that the left common carotid artery and internal carotid artery were occluded, the left subclavian artery was narrow, and the right internal carotid artery was extremely narrow. After admission, the patient was subjected to a high-resolution magnetic resonance examination of the brain, which suggested that there were multiple acute infarction lesions under the left cerebral cortex and beside the lateral ventricle, following which hypoperfusion infarction was considered (Figure 1). A digital subtraction angiography showed that the left common carotid artery was occluded, the left subclavian artery was extremely narrowed (∼90%), the left vertebral artery opening was narrowed (∼50%), the left posterior cerebral artery supplied blood to the left middle cerebral artery supply area through the pia mater, the V1 segment of the right vertebral artery was occluded, the C1 segment of the right internal carotid artery was extremely narrowed (∼80%), and the right anterior communication was open (Figure 2).

A combination of the characteristics of medical history and imaging revealed that the cause of acute cerebral infarction was hypoperfusion resulting from left common carotid artery occlusion and insufficient collateral compensation. However, considering that the occlusion time of the left common carotid artery of the patient could not be determined, the occlusion position of the common carotid artery was found to be low, and therefore, surgery for left common carotid artery occlusion was thought to be risky and time-consuming. Therefore, the surgical plan that was proposed was the implantation of the C1 segment stent of the right internal carotid artery and that of the left subclavian artery. At the same time, considering that the vessels on both sides of the patient are chronically severe stenosis, the risk of CHS after stent implantation is high. So that the blood pressure levels should be strictly monitored and controlled after the operation, and attention should be paid to the possibility of carotid sinus reaction, reflex hypotension, and slow heart rate. There is no difference between CAS and CEA in terms of preventing long-term risk and long-term death risk of ipsilateral long-term stroke, severe stroke, or perioperative death, and the risk of cranial nerve injury during the perioperative period of stent implantation is low. After the patient and his family were fully informed about the risks and precautions related to CAS and CEA, their consent was obtained after they expressed their understanding and strongly requested stent implantation.

**Figure 3:**
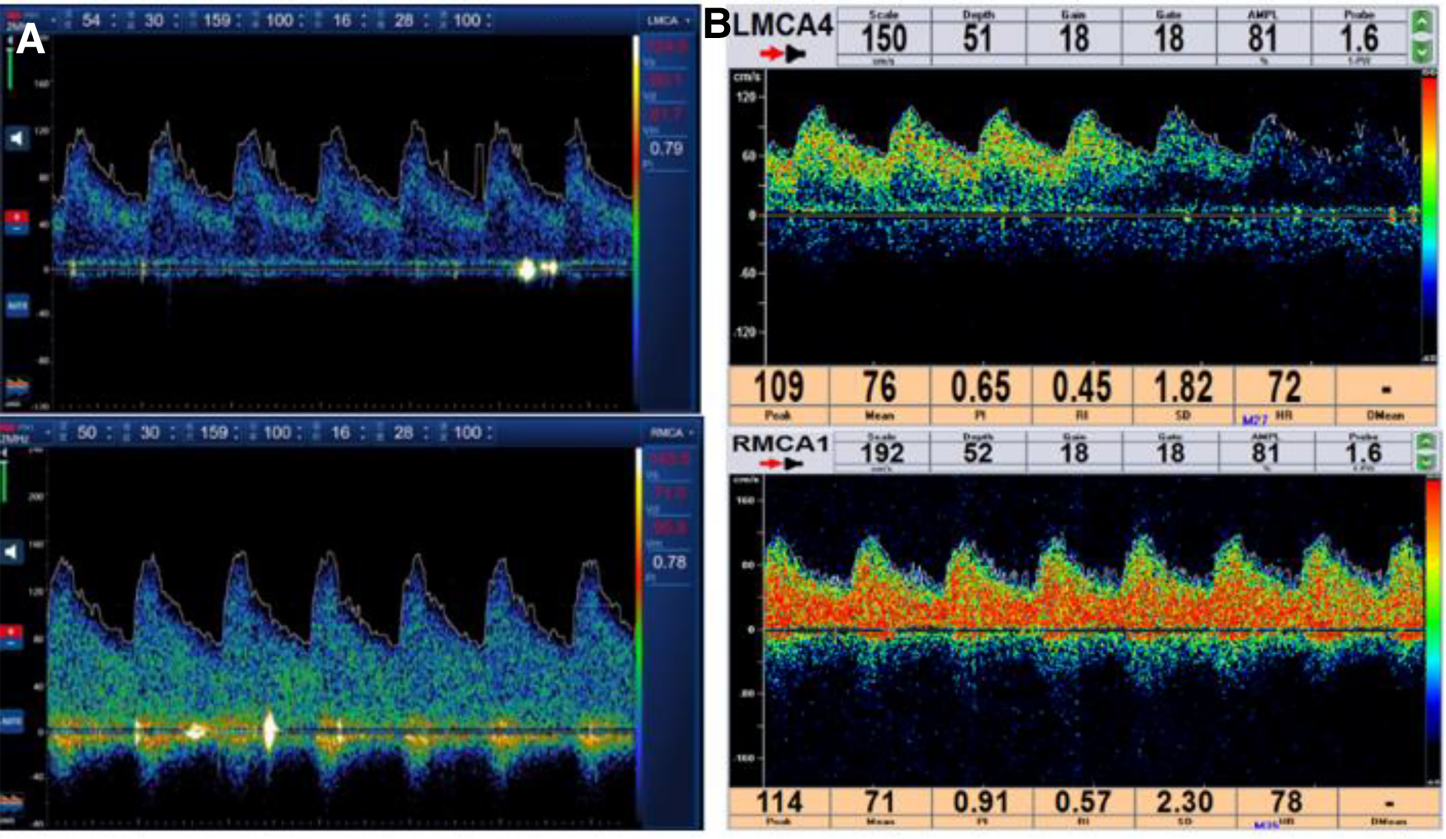
(**A**) Spectrum results of the bilateral middle cerebral artery detected by TCD after CAS. (**B**) Spectrum results of the bilateral middle cerebral artery detected by TCD before the operation. TCD, transcranial Doppler ultrasound; CAS, carotid artery stent implantation.

**Figure 4:**
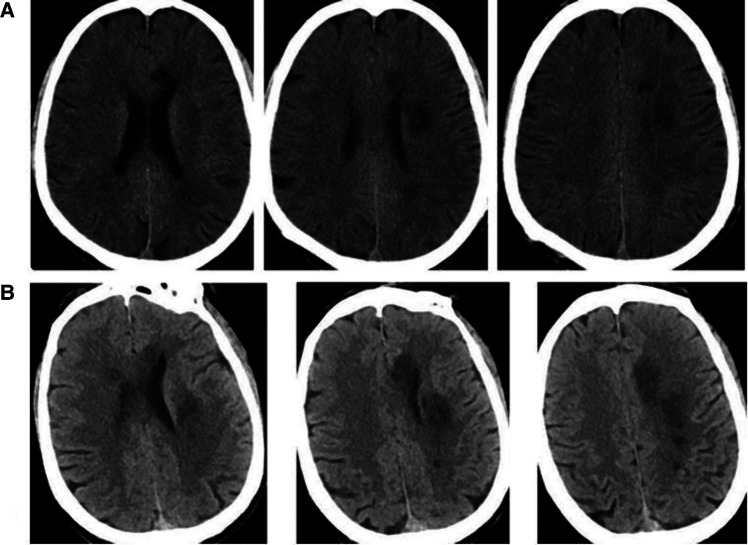
(**A**) Brain CT images were reexamined on the day after CAS. (**B**) On the third day after CAS, the brain CT image was reexamined (the low-density focus showing a greater enlargement than before). CAS, carotid artery stent implantation.

**Figure 5:**
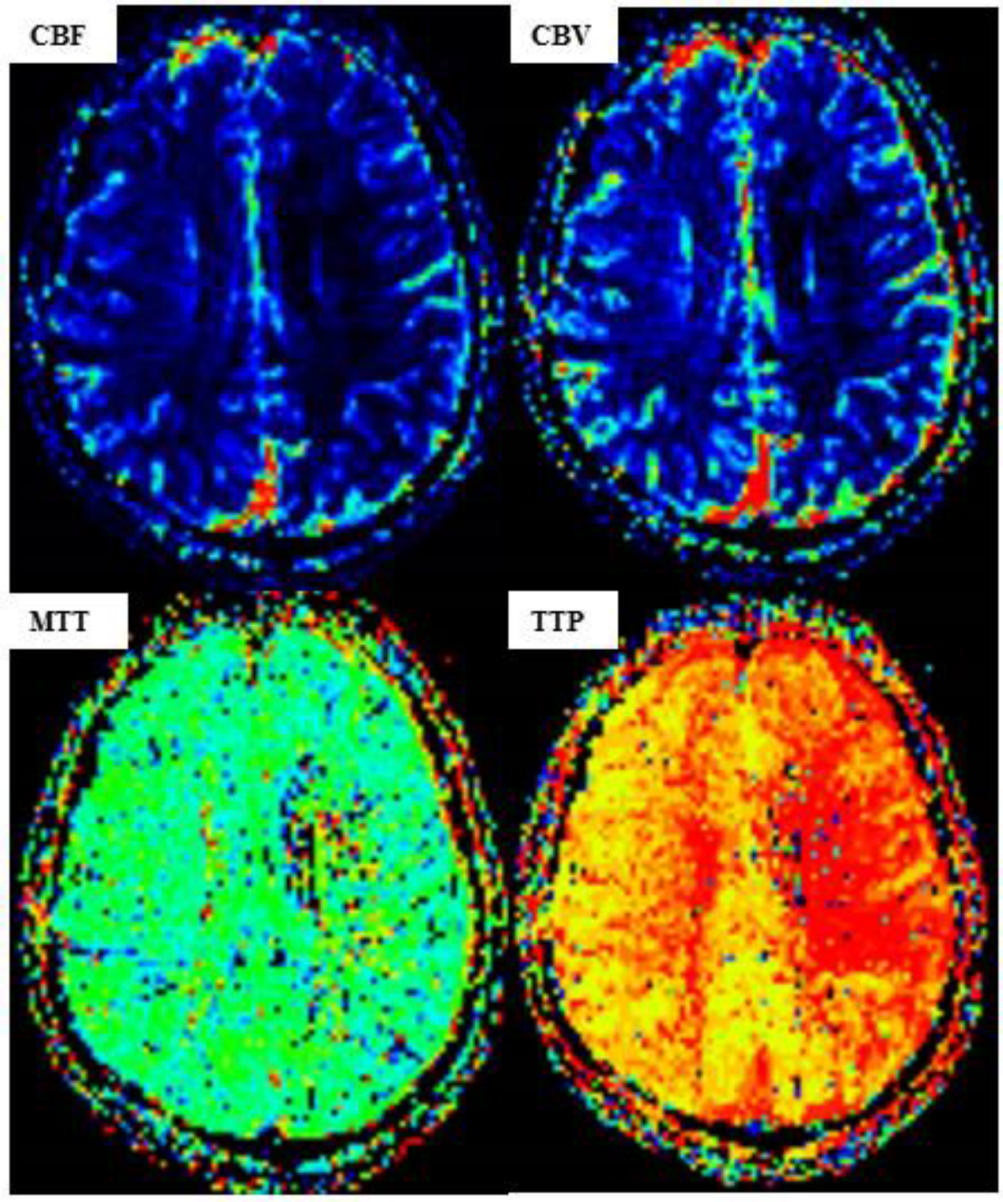
MR perfusion-weighted imaging (PWI) after carotid artery stent implantation (CAS). [PWI showing a lower cerebral blood volume (CBV) and cerebral blood flow (CBF) in the left hemisphere than those in the right hemisphere, while the time to peak (TTP) and mean transit time (MTT) are significantly higher.]

**Figure 6:**
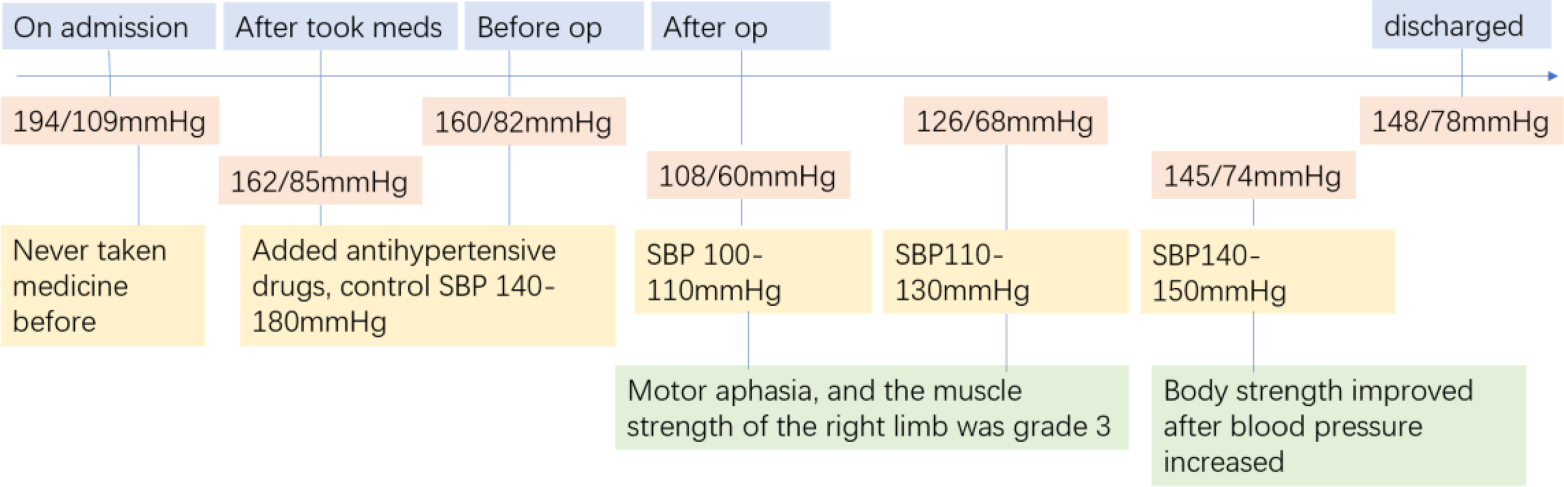
Timeline with relevant blood pressure data.

The high-risk factors of CHS include long-term hypertension, carotid stenosis of the treatment side > 90%, and occlusion or stenosis of the contralateral carotid artery > 80%. Some studies indicate that for high-risk patients, it is recommended to control blood pressure < 120/80 mmHg after CAS. On the 10th day of admission, we performed stent implantation of the C1 segment of the right internal carotid artery and left subclavian artery for the patient. ASA 100 mg, plavix 75 mg, and nifedipine controlled-release tablets of 30 mg were given on the day of the operation. The embolic protective device used during the operation was SpiderFX 6.0 mm (EV3) protective umbrella. Transient vagal reaction occurred during stent implantation and balloon dilation, the heart rate and blood pressure decreased, and 0.5 mg of atropine was immediately injected intravenously, following which the patient's heart rate and blood pressure returned to normal, and ECG and blood pressure monitoring were done in real time. Heparin (800 IU ivgtt) was used three times after the operation. Low-molecular-weight heparin (5000 IU ih q12h) was used for 3 days after the femoral artery sheath was removed. Collateral circulation improved after the operation. Then, the patient was transferred to the NICU, and the systolic blood pressure was controlled at a range of 100 and 110 mmHg. Several hours later, the patient developed motor aphasia, and the muscle strength of the right limb was grade 3. A bedside TCD examination and brain CT examination were performed immediately. TCD showed that the flow velocity of the middle cerebral artery had changed little before and after the operation (Figure 3). The brain CT showed no hemorrhage and obvious swelling of the cortex, and CHS was preliminarily excluded (Figure 4A). On the second day, MRI cerebral perfusion was done, which revealed a decrease in perfusion in the left frontotemporal lobe which was more severe than that before surgery (Figure 5). It was considered that the patient’s condition may be aggravated by hypoperfusion cerebral infarction due to the strict control of blood pressure after the operation. He was immediately treated with blood volume supplementation and pressure boosting. During this process, it was found that the weakness of the right limb was aggravated when the blood pressure was decreased (the systolic pressure was lower than 120 mmHg), and the weakness of the right limb was relieved when the blood pressure was increased (the systolic pressure was greater than 150 mmHg). On the third day after the operation, the patient regained his consciousness, but suffered from aphemia, and the muscle strength of the right limb was grade 0. Upon a reexamination of the brain CT, it could be seen that there was an enlargement of the low-density focus beside the left lateral ventricle (Figure 4B). After providing active medical treatment and rehabilitation physiotherapy, the patient was discharged from the hospital on the 10th day after the operation. At the time of discharge, he was conscious, but continued to have aphemia, and the muscle strength of the right limb was grade 3. A timeline with relevant blood pressure data is shown in Figure 6.

## Discussion

Ischemic stroke accounts for more than 80% of all stroke, and internal carotid artery stenosis is a high-risk factor for ischemic stroke. Symptomatic carotid stenosis ≥ 50% or asymptomatic carotid stenosis ≥ 75% is an indication for CAS. The postoperative complications of CAS include CHS, cerebral infarction, vascular injury, carotid sinus reaction, and stent restenosis.

In this case, after strict preoperative evaluation, we decided to first implant the right internal carotid artery stent and then open the left common carotid artery at a selected time, mainly because it is difficult to open the left common carotid artery, the operation time is long, and the risk of CHS is high. At present, there is no case report of severe stenosis on one side of the internal carotid artery and occlusion and simultaneous opening of the contralateral common carotid artery either at home or abroad.

Some studies have shown that the treatment side carotid artery stenosis is more than 90% and the contralateral carotid artery stenosis is more than 80% or occlusion, accompanied by hypertension, which are high risk factors for CHS.

The methods of examination of CHS include transcranial Doppler ultrasound (TCD), single photon emission computed tomography (SPECT), CT perfusion, and perfusion-weighted imaging. TCD is a convenient, fast, and bedside detection technology, which is of great value in the evaluation of CHS. TCD can measure the blood flow velocity, peak blood flow velocity, and pulsatility index of the middle cerebral artery and can be used to evaluate ipsilateral cerebral blood flow perfusion. A 1.5-fold or 2.0-fold increase in the mean flow velocity of the middle cerebral artery after surgery compared with that before surgery indicates a highly suspected case of CHS.

Prevention of CHS includes a complete preoperative evaluation, selection of anesthesia mode, and strict postoperative blood pressure control. Most of the current studies on CAS suggest that the blood pressure levels after surgery should be kept at <140/90 mmHg. For high-risk patients, some studies suggest that it is reasonable to control the postoperative blood pressure below 120/80 mmHg.

Considering the high risk of CHS after CAS in our patient, his postoperative blood pressure was strictly controlled below 120/80 mmHg, but there was a rare hypoperfusion infarction after the operation. The possible reason for this is that although the contralateral internal carotid artery is opened and the right to left branches of the anterior communicating artery are compensated and improved, these are still insufficient to offset the decrease of cerebral blood flow caused by the decrease of blood pressure. It is considered that the patient is in a state of intracranial hypoperfusion for a long time, is sensitive to blood pressure, and has poor long-term blood pressure control. The increase of blood pressure may be an adaptive response of the body to hypoperfusion.

Cerebral infarction after CAS is often seen in the distal vessels embolized by the shedding of microemboli. Some studies have suggested that the incidence of cerebral micro infarction after CAS is 26%—70.8%. The risk factors include old age, ulcer plaque, long segment disease, smoking history, plaque calcification, aortic arch anatomical characteristics, and internal carotid and common carotid artery angles. However, with the application of a distal brain protection device, the complication of distal embolic infarction is significantly reduced. Hypoperfusion infarction after CAS is very rare. The cerebral infarction in this case is presumed to be hypoperfusion infarction caused by strict postoperative blood pressure control. Therefore, for patients with severe stenosis of the carotid artery with hypoperfusion infarction and contralateral common carotid artery occlusion before CAS, it may be more advantageous to control blood pressure to  < 140/90 mmHg or to a higher level after CAS.

Some studies have shown that the degree of endothelial function damage caused by different interventional treatment methods varies . Other studies have shown that the location, duration, and degree of endothelial injury caused by different types of scaffolds are different, and many studies at home and abroad hold different views on this. Stent implantation can cause a certain degree of endothelial damage. By reducing the concentration of nitric oxide in the relevant areas and interfering with its distribution, the nitric oxide concentration near the stent will become relatively low, further leading to stent restenosis and thrombosis (1). The endothelial damage caused by bare metal scaffolds belongs to the category of mechanical damage. The metal scaffolds will peel off the adventitia and media of the blood vessels and eventually deposit many exfoliated endothelial cells in the vessels, thus causing thrombosis. In our case, the symptoms of limb weakness significantly improved when the blood pressure increased after the operation, and the symptoms were not persistent, so the possibility of thrombosis and cerebral infarction caused by endothelial injury was small.

Due to the presence of baroreceptors in the carotid sinus, the reflective heart rate and blood pressure will drop during stent implantation or balloon expansion. Some studies have found that the probability of sinus bradycardia and postoperative hypotension is as high as 80%. The incidence of carotid sinus reaction is as high as 36.7%. Strasberg et al. (2) defined carotid sinus reaction (CSR) as cardiac arrest ≥ 3 S (or cardiac rhythm decline ≥ 50%) and/or systolic blood pressure ≤ 90 mmHg (1 mmHg = 0.133 kpa) (or systolic blood pressure decline ≥ 50 mmHg). If not handled properly, it may cause hypoperfusion, new cerebral infarction, TIA, or even cardiac arrest in patients with severe conditions and bring disastrous consequences to them. In our patient, the systolic pressure was not lower than 100 mmHg during and after the operation, so the possibility of hypoperfusion cerebral infarction caused by carotid sinus reaction was small.

In-stent restenosis (ISR) after CAS refers to the loss of lumen in the whole process of stent implantation and/or at 5 mm segments at both ends of the stent, resulting in a lumen stenosis rate of ≥ 50%. It is a common complication, with an incidence rate ranging from 4.7% to 12.5% (3). ISR not only affects the long-term prognosis of patients and reduces the quality of life, but also easily causes the recurrence of ischemic stroke, resulting in severe economic losses to patients and consequent burden to their families. The risk factors of stent restenosis after CAS include female sex, age, diabetes, dyslipidemia, use of a closed cell stent, carotid endarterectomy, and cervical radiotherapy (3). In our patient, the above-mentioned risk factors hardly exist, and the possibility of their occurrence is small.

The MRI of the patient at admission revealed a partial subcortical watershed infarction, indicating the existence of perfusion failure in the cerebral artery terminal region. The autoregulation ability of the brain is impaired in this area with reduced perfusion, which may be related to the reduction of collateral cerebral blood vessels (4). To prevent the occurrence of CHS after the operation in our patient, his blood pressure was controlled at 100–120 mmHg, resulting in further impairment of the patient's brain autoregulation function, perfusion-dependent accumulation of cytotoxic metabolites and tissue acidosis, and further aggravation of hypoperfusion cerebral infarction. This shows that a strict control of blood pressure is not necessarily beneficial to patients. A perfusion examination and a normal blood pressure level test should be fully completed before the operation, in order to predict the possibility of CHS after CAS and to control postoperative blood pressure levels. At present, we speculate that a level <140/90 mmHg or higher may be more favorable to our patient.

## Conclusion

In clinical practice, in general, we should consider the possibility of the occurrence of hyperperfusion syndrome after interventional therapy, because an increase in the patient's blood pressure level before surgery may be the result of long-term compensation of the body. After surgery, we should not simply control the blood pressure to a low level but should develop a personalized treatment plan based on the patient's blood pressure level that existed before surgery.

## Data Availability

The original contributions presented in the study are included in the article/Supplementary Material; further inquiries can be directed to the corresponding author.
